# Macrophage-derived CXCL8 as a mediator of inflammatory attacks in Meniere’s disease

**DOI:** 10.3389/fimmu.2026.1683879

**Published:** 2026-03-06

**Authors:** Lu Peng, Boyu Zhu, Yongpeng Li, Ying Lan, Xiaolin Zhan, Xiao Pan, Shiliao Li, Shihua Yin

**Affiliations:** Department of Otorhinolaryngology Head and Neck Surgery, The Second Affiliated Hospital of Guangxi Medical University, Nanning, Guangxi, China

**Keywords:** chemotaxis, CXCL8, macrophage, Meniere’s disease, neuroinflammation

## Abstract

**Background:**

Ménière’s disease (MD) is a complex disorder whose pathogenesis extends beyond endolymphatic hydrops to involve dysregulated immune responses. While a subset of patients exhibits a “low-cytokine phenotype” during remission, the mechanisms underlying the transition to acute inflammatory attacks triggered by environmental factors remain poorly understood.

**Methods:**

We employed an integrative multi-omics approach to explore the immune microenvironment of MD. This included bioinformatic analysis of differentially expressed genes (DEGs) from GSE109558, featuring PBMCs from MD patients and healthy controls stimulated with Aspergillus or Penicillium. Protein-protein interaction (PPI) networks, immune infiltration analysis, and single-cell RNA sequencing (GSE269117) were utilized to identify hub genes and cellular interactions. Key findings were validated in an independent cohort through measurement of serum cytokines, *in vitro* macrophage stimulation assays, and immunofluorescence staining.

**Results:**

Bioinformatic analysis revealed a latent hyperinflammatory potential in MD PBMCs, which was unmasked upon fungal challenge, showing significant enrichment in neutrophil chemotaxis and NF-κB pathways. We identified 20 hub genes, with CXCL8 emerging as a top candidate. Single-cell sequencing and CellChat analysis pinpointed macrophages as the dominant source of CXCL8 and key orchestrators of intercellular communication, notably via the ALCAM-CD6 pathway with T cells. *In vitro* verification confirmed this macrophage-driven inflammatory cascade response. Under the stimulation of LPS/β -glucan, the level of CXCL8 secreted by macrophages in MD patients increased (p < 0.01), while there was no difference before and after stimulation in the healthy control group. Serum levels of CXCL8, IL-6, and IL-17A were also significantly elevated in MD patients during attacks.

**Conclusion:**

Our findings support a novel “hypoimmune-hyperinflammatory switch” model in MD, wherein macrophages play an important role in initiating and amplifying inflammatory responses to environmental triggers via CXCL8 production and cellular crosstalk. This refined understanding of the immune axis in MD provides a foundational basis for developing targeted immunomodulatory therapies.

## 1 Introduction

Meniere’s disease (MD) is one of the most common peripheral vestibular disorders. It is characterized by recurrent vertigo, fluctuating hearing loss, aural fullness, and tinnitus. The global prevalence of MD ranges from 3.5 to 513 per 100,000. It is rare in children but more common in middle-aged adults. As a chronic disabling disease, MD can be unpredictable in terms of symptom occurrence and severity. This uncertainty about when symptoms will occur seriously impacts patients’ quality of life and increases the healthcare burden (1). The clinical management of MD remains limited and primarily focuses on symptomatic treatments such as diuretics and betahistine. However, these treatments do not address the root causes of the disease or prevent its recurrence (2). This highlights the urgent need for new approaches that target the underlying causes of MD.

The etiology of MD is complex and involves a combination of genetic, environmental, immunological, and anatomical factors (3).While the exact pathogenesis of MD is not fully understood, but endolymphatic hydrops is a main pathological feature. However, it cannot fully explain the episodes and clinical heterogeneity of MD (3, 4). Familial aggregation occurs in approximately 5–20% of cases in European populations. Mutations have been found in genes such as OTOG, MYO7A, and TECTA, which are critical for inner ear structural integrity and function (5). Sporadic MD, unlike monogenic familial forms, involves polygenic contributions, especially in immune regulatory pathways. Notable examples include TLR3, which plays a role in antiviral innate immunity and inner ear inflammation, and MEFV, which regulates IL-1β-mediated inflammatory processes (6). These variations indicate a substantial predisposition to MD, while environmental factors may trigger pathological processes in susceptible individuals. The disease can be triggered by several factors, including autoimmunity, infections, trauma, allergies or obstruction of the endolymphatic duct and/or capsule (1, 3).

The clinical heterogeneity of MD is reflected in its subtypes, which include sporadic classical MD; sporadic delayed MD; familial MD; sporadic MD with migraine; and sporadic MD with autoimmune disease (4). Despite this heterogeneity, all subtypes share the common endpoint: the development of EH. Several theories have been proposed to explain cochleovestibular dysfunction and the occurrence of acute attacks. These include membranous labyrinth rupture leading to potassium toxicity affecting hair cells and neurons; hydromechanical fluctuations causing direct distortion of sensory structures; and ischemic episodes resulting from hemodynamic instability and ion channel dysfunction. Additionally, a growing body of evidence suggests that acute inflammatory responses can lead to temperature fluctuations within the confined space of the inner ear. This localized inflammation theory provides a critical link between immune dysregulation and the symptoms of MD by directly impairing vestibular hair cell and nerve function (3).

Recent research has focused on the complex immunological mechanisms associated with MD. Increased levels of proinflammatory cytokines, including TNF-α, IL-6, and IFN-γ, have been activated in the endolymphatic sac (ES), while systemic inflammatory markers remain unchanged in serum (7). A subset of MD patients (42%) exhibits a “low-cytokine phenotype” during quiescent phases. This phenotype is characterized by suppressed immune activity (8). Furthermore, around 30–40% of MD patients have comorbid allergic disorders, and a subset shows cytokine profiles that are either high or low (e.g., IL-1β) (9, 10), indicating immune heterogeneity.

The inner ear contains macrophages and lymphocytes that process antigens and trigger local immune responses. Environmental exposures, such as fungal components (e.g., Aspergillus, Alternaria) as well as viral or bacterial components, can contribute to the onset and progression of MD (10–13). Studies show that air pollutants like SO_2_, NO_2_, and PM2.5 are associated with hospital visits for MD patients (12). Components from fungi (e.g., Aspergillus, Penicillium) or bacteria can trigger activation of the NF-κB pathway in monocytes and macrophages. Activation of this pathway transforms low-level immune responses triggered by environmental factors into chronic inflammation, causing peripheral blood mononuclear cells (PBMCs) from MD patients to release large amounts of proinflammatory cytokines such as TNF-α and IL-1β (8, 14). These observations suggest that MD may involve a “hypoimmune-hyperinflammatory switch”, whereby during clinically quiescent periods, a state of reduced immune vigilance (hypoimmune state) may prevail. This state allows minor environmental triggers—including lifestyle factors (e.g., sleep deprivation, high salt intake), subclinical infections, allergens, or pollutant exposure—to initiate inflammatory reactions. These reactions are disproportionate and intense, constituting a hyperinflammatory state that leads to symptom recurrence. Although inflammation contributes to MD, the precise roles of cellular orchestrators, particularly macrophages, and the cytokine CXCL8 in this ‘switch’ paradigm are still poorly understood.

To validate this hypothesis, the study seeks to investigate the molecular mechanisms and characteristics of the immune microenvironment associated with MD. This will be achieved through a thorough analysis of gene expression, functional enrichment, the construction of a protein-protein interaction (PPI) network, and single-cell analysis. By utilizing advanced bioinformatics approaches, including differential gene expression profiling and immune landscape assessment, we seek to identify key pathways and interactions that contribute to the pathophysiology of MD. Finally, we isolate peripheral blood monocytes from MD patients during acute attacks and healthy controls, differentiate them into macrophages, and stimulate them with LPS and β-glucan to mimic innate immune triggers. CXCL8 secretion was quantified via ELISA, and co-expression of CD68 (macrophage marker) and CXCL8 was visualized using immunofluorescence. Combining multiomics profiling with targeted experimental validation, we elucidate the immune dynamics underlying MD and identify novel therapeutic targets. Our integrated approach provides a nuanced perspective on MD pathogenesis, aligning with its multifactorial nature while highlighting actionable immune mechanisms.

## 2 Materials and methods

### 2.1 Study design at a glance

**Figure 1:**
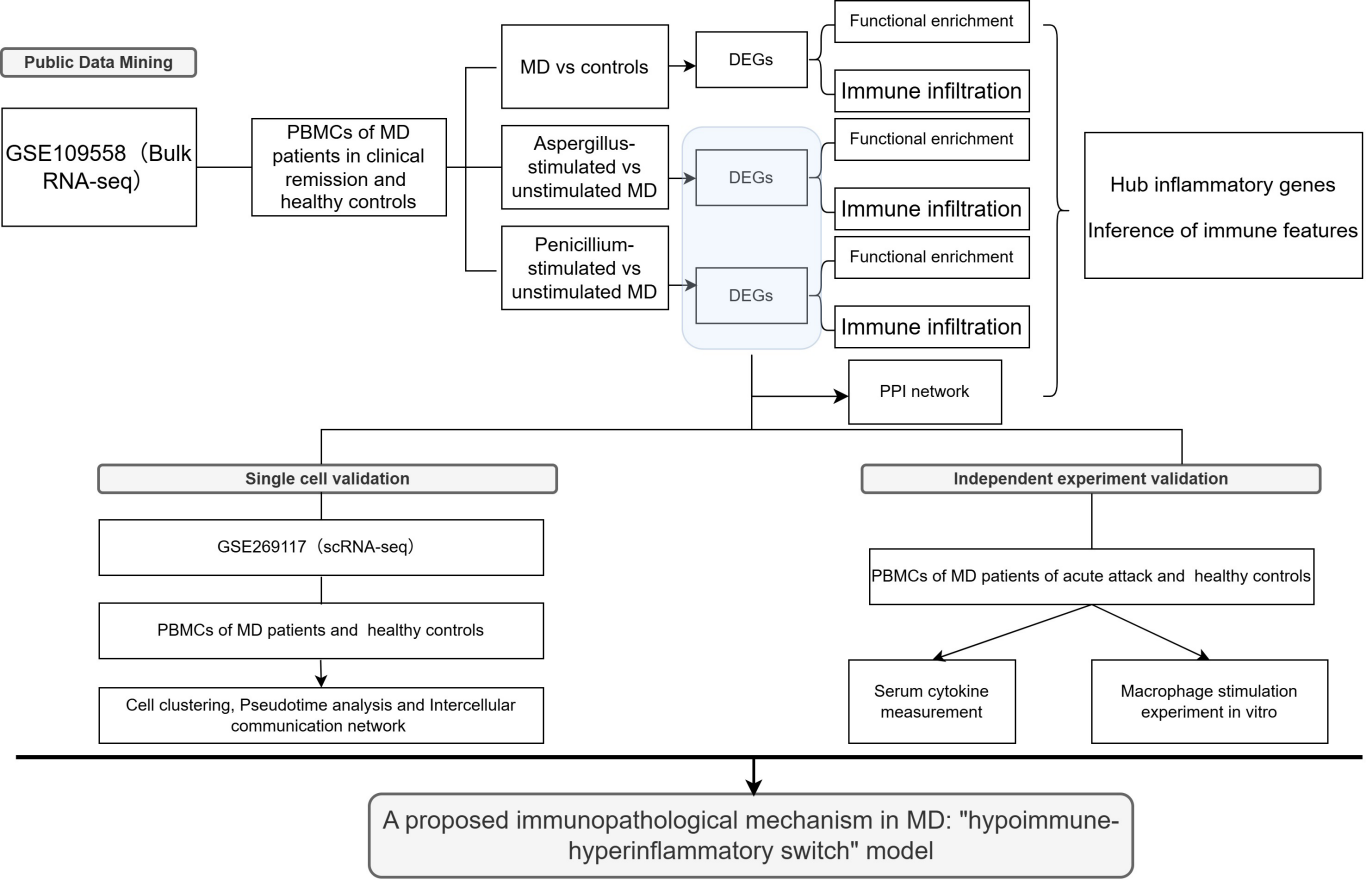
The flowchart of this study. DEGs, differentially expressed genes; PBMCs, peripheral blood mononuclear cells; MD, Meniere’s disease; PPI, protein-protein Interaction Networks.

**Figure 1** shows the flowchart of our study. We utilized dataset GSE109558 to analyze gene expression in PBMCs from MD patients in clinical remission and in PBMCs from healthy controls. The expression of genes was compared in patients with MD treated with Aspergillus or Penicillum fungal mixtures and MD untreated in this dataset. The validation of serum cytokine measurements was made by comparing MD patients with controls. Then we investigated the single cell dataset GSE269117 for cellular sources of hub inflammatory genes. *In vitro*, PBMCs were differentiated into macrophages and stimulated with LPS and β-glucan of MD patients during the acute attack and controls.

### 2.2 Exploration cohorts acquisition and processing

Two datasets were obtained from publicly available sources. The GSE109558 dataset was downloaded from the NCBI GEO databases (https://www.ncbi.nlm.nih.gov/geo/) (8). This dataset utilized the GPL10558 platform (Illumina HumanHT-12 V4.0 expression BeadChip). The experiment compared gene expression in PBMCs of 15 MD patients and 11 healthy controls. In some patients and healthy controls, four equal parts of a mixture of Aspergillus species (oryzae, repens, niger and terreus), and 4 equal parts of a mixture of Penicillium species (brevicompactum, expansum, notatum and roqueforti) were added. Comparisons were conducted between untreated MD patients and untreated healthy controls. Additionally, comparisons were made between Aspergillus- or Penicillium-treated MD patients and untreated MD patients.

The single-cell sequencing dataset GSE269117 was used to analyze specific molecular characteristics in patients with Ménière’s disease (15). This dataset includes 5 vestibular migraine patients, 6 healthy controls, 5 migraine patients, and 8 MD patients. Sample GSM8306612 was excluded due to missing column data. Therefore, 5 healthy controls and 8 MD patients were selected for further analysis.

Sample details in both datasets are as follows. Whole blood samples were collected from patients with MD and healthy controls. In GSE109558, no subject had been received steroid therapy in the past three months, and all subjects had negative skin prick tests. All patients were in the remission phase of MD. In GSE269117, no subject had been diagnosed with allergies or autoimmune diseases. Sample details for each dataset are provided in **Table 1**.

**Table 1:** Details of exploration cohorts.

Dataset	Platform	Group	Sample
GSE109558	GPL10558 (Illumina HumanHT-12 V4.0 expression beadchip)	control	n = 5
		Aspergillus stimulation in control	n = 3
		Penicillium stimulation in control	n = 3
		Meniere disease	n = 7
		Aspergillus stimulation in Meniere’s disease	n = 4
		Penicillium stimulation in Meniere’s disease	n = 4
GSE269117	GPL33758 (HiSeq X Five)	control	n = 6
		Meniere disease	n = 8
		Migraine	n = 5
		vestibular migraine	n = 5

Quality control and filtering for both datasets were performed as follows. First, the two datasets were annotated and standardized before proceeding to the next step of analysis. In GSE269117, a Seurat object was generated utilizing version 5.3.0 of the Seurat package in R, specifically for the purpose of conducting quality control (16). Cells with Unique Molecular Identifier (UMI) counts ranging from 200 to 5,000 were retained; low-quality cells with proportions of UMIs derived from mitochondrial genes greater than 20% were filtered out. The remaining cells were used for subsequent analysis.

### 2.3 Independent cohort acquisition and processing

The Second Affiliated Hospital of Guangxi Medical University Ethics Committee approved the protocol for this study (No: 2024-KYL(004)), and informed consent was obtained from all participants.

From January 2024 to September 2025, thirteen inpatients diagnosed with MD and admitted to our hospital were recruited. The diagnosis of MD in these patients was based on the Clinical Practice Guideline of Meniere’s Disease (1). All patients were in the attack stage of the disease. Thirteen healthy volunteers matched for age and gender were selected as the control group. None of the subjects had any diagnosed allergies or autoimmune diseases. The controls were excluded if they had otological diseases, a history of vertigo, migraine, or other related neurological or cardiovascular conditions. Details for each patient are provided in **Supplementary Table S1**. A subset of this cohort was utilized for specific experiments: serum samples from 8 MD patients and 8 matched controls were used for cytokine measurement, while PBMCs isolated from another 5 MD patients and 5 controls were used for *in vitro* assays.

### 2.4 PBMC isolation, differentiation, and stimulation

The exploration cohort data (GSE109558 and GSE269117) were derived from human PBMCs. For the GSE109558 dataset, PBMCs were isolated from whole blood using density gradient centrifugation. These cells were then cultured and stimulated with fungal antigens for subsequent gene expression analysis (8). The GSE269117 single-cell sequencing dataset was generated from purified PBMC nuclei using the 10x Genomics Multiome platform (15). Five milliliters (mL) of fasting venous blood were collected from each subject. The blood samples from sixteen participants (8 MD patients and 8 controls) were collected, and serum was isolated by centrifugation at 3500 rpm for 10 minutes. Collected serum was stored at -80 °C until analysis. PBMCs were isolated from fresh blood samples from other ten participants (5 MD patients and 5 controls). The cells were washed twice with PBS (P1020, Solarbio, Beijing, China) and then resuspended in complete RPMI-1640 medium. To differentiate monocytes into macrophages, PBMCs were seeded and cultured for 5 days in medium supplemented with 2 μL GM-CSF (K20051, Shanghaiyuanye, Shanghai, China), with the medium changed on day 3. Differentiation efficiency was confirmed by flow cytometric analysis showing that more than 85% of the cells expressed the surface marker CD14 (E-AB-F1209C, Elabscience, Wuhan, China). Following successful differentiation, the macrophages were stimulated for 24 hours under two distinct conditions: a negative control group, where cells were cultured in RPMI 1640 medium (L103-500, BDBIO, Shanghai, China) supplemented with 10% FBS (20230228, Hycezmbio, Wuhan, China); and a compound stimulation group, where cells were treated with 100 ng/mL lipopolysaccharide (LPS, L8880, Solarbio, Beijing, China) and 10 μg/mL β-glucan (IG4240, Solarbio, Beijing, China).

### 2.5 Screening for differentially expressed genes

Aspergillus and Penicillium are common environmental fungi that can trigger immune responses, especially allergic diseases (17). We verified the basal inflammatory state in the remission phase of MD by comparing DEGs in PBMCs from MD patients and healthy controls. The immune response to different environmental fungi in the remission phase of MD patients was demonstrated by comparing DEGs in PBMC stimulated or unstimulated with Aspergillus or Penicillium. Analysis was conducted using the limma package (18) in R software. Genes exhibiting an absolute log fold change greater than 0.5 and an adjusted p-value of less than 0.05 were classified as differentially expressed. The outcomes of the differential expression analysis were subsequently illustrated through volcano plots.

### 2.6 Functional enrichment analysis

Gene Ontology (GO) and Gene Set Enrichment Analysis (GSEA) were performed using the clusterProfiler R package (19). The gene annotation data were sourced from the org.Hs.eg.db R package (20). In the GO analysis, three comprehensive categories were examined: Biological Process (BP), Cellular Component (CC), and Molecular Function (MF). Within each category, differentially expressed genes (DEGs) with p-values less than 0.05 and q-values below 0.1 were deemed significant. We treated these terms as significant. In GSEA, the analysis was focused on gene sets related to innate immunity and CXCL8-mediated biological processes. Significance thresholds were set as p-value < 0.05 and FDR < 0.25. The top 10 enriched pathways were visualized using the ggplot2 (21) packages to generate dot plots.

### 2.7 PPI network construction and hub genes screening

Co-expressed genes were identified by intersecting DEGs from Aspergillus-stimulated versus unstimulated MD groups with those from Penicillium-stimulated versus unstimulated MD groups. We used the STRING database (https://cn.string-db.org/) to establish the PPI network. The parameters were set as follows: the organism was set to ‘Homo sapiens’, the minimum interaction score was set to 0.7, and disconnected nodes were omitted. During the visualization of the PPI network, key modules were identified using the MCODE algorithm within the cytoHubba plugin (22). The top 20 genes with the highest connectivity degree in the network were selected as hub genes.

### 2.8 Expression analysis of hub genes

The expression of the 20 hub genes (identified from the PPI network) was visualized using box plots across the four groups: healthy controls, MD patients, Aspergillus- and Penicillium-stimulated MD patients. We compared MD patients versus healthy controls, Aspergillus-stimulated versus unstimulated MD patients, and Penicillium-stimulated versus unstimulated MD patients.

### 2.9 Immune landscape analysis

We assessed the immune microenvironment across four groups: MD patients versus healthy controls, Aspergillus-stimulated versus unstimulated MD patients, and Penicillium-stimulated versus unstimulated MD patients. Immune cell infiltration levels were evaluated using single-sample Gene Set Enrichment Analysis (ssGSEA) based on Bindea etal. (23) and CIBERSORTx (LM22) (24). Correlations between 20 hub genes, identified from the PPI network, and immune cells were analyzed and visualized using the GSVA (25), CIBERSORTx (26), and corrplot (27) packages in R 4.3.

### 2.10 Serum cytokine in patients with MD

The concentrations of 12 pro-inflammatory cytokines (IL-1β, IL-2, IL-4, IL-5, IL-6, IL-8, IL-10, IL-12P70, IL-17A, TNF-α, IFN-γ, and IFN-α) were simultaneously determined by immunofluorescence. The assay was conducted precisely following the manufacturer’s guidelines included with the Cytokine Combined Detection Kit (Cellgene Biotech, Jiangxi, China). Each sample was measured in triplicate, and the average value was used for statistical analysis.

### 2.11 Single cell data analysis

Principal Component Analysis (PCA) was performed utilizing highly variable genes, resulting in the selection of the top 30 principal components for the purpose of clustering analysis. Subsequently, cells were clustered at a resolution of 0.8, followed by cell annotation using the SingleR package in conjunction with CellAtlasData (28). Based on highly variable genes (dispersion ≥ 0.5 and average expression ≥ 0.1), a CellDataSet was constructed using the cell clusters obtained from clustering as input. The DDRTree algorithm was employed for the purpose of reducing dimensionality, while the order Cells function was utilized to explore the differentiation trajectory of the cells. Expression trends of CXCL8 along the pseudotime axis were visualized using the plot_cell_trajectory function. Finally, the CellChat package was used to analyze the intercellular communication network (29). A database of ligand-receptor interactions was established, drawing from known interactions pertinent to the specified cell types.

For visualization of gene expression across cell types and conditions, we focused on a targeted set of genes selected based on prior analyses. From the top 20 hub genes identified in the PPI network, we chose seven genes (IL6, IL10, CCL3, CCL19, CXCL1, CXCL8, CCL20) that showed significant correlations with immune cell infiltration in the Aspergillus- and Penicillium-stimulated groups. We also included cytokines (IL6, CXCL8, IL4, IL17A) that were significantly differentially expressed in our serum validation data. Additionally, to provide broader immunological context, we incorporated CXCL12, a canonical homeostatic chemokine relevant to innate immunity. The netVisual_circle function was used to visualize communication intensity among cell types. The interaction patterns of key ligand-receptor pairs were displayed separately, while communication probabilities for various signaling pathways were calculated in prior analysis steps.

### 2.12 Enzyme-linked immunosorbent assay

The concentration of CXCL8 in the cell culture supernatant was quantified using a commercial human CXCL8/IL-8 ELISA Kit (HM10222, Bioswamp, Wuhan, China), according to the manufacturer’s protocol.

### 2.13 Immunofluorescence

Cells were fixed for 15 minutes at room temperature, permeabilized with 0.2% Triton X-100 Prepared with 1xPBS) for 15 minutes, and blocked with 5% goat serum (Prepared with 1xPBS) for 30 minutes at 37°C. Subsequently, sequential immunofluorescence staining was performed. The cells were first incubated with an anti-CD68 primary antibody (PAB43782, 1:100, Bioswamp, Wuhan, China) overnight at 4°C, followed by an HRP-conjugated secondary antibody (Ab150079, Abcam, Shanghai, China) for 50 minutes at room temperature. The signal was amplified using an iF647-tyramide working solution for 10 minutes. After thorough washing, the cells were incubated with an anti-CXCL8/IL-8 primary antibody (PAB59641, 1:200, Bioswamp, Wuhan, China) overnight at 4°C, followed by an HRP-conjugated secondary antibody (ab150077, Abcam, Shanghai, China) and subsequent signal amplification with an iF488-tyramide working solution. Nuclei were counterstained with DAPI. Imaging was performed using a fluorescence microscope, and the percentage of CD68^+^ double-positive cells was quantified with ImageJ software.

### 2.14 Statistical analysis

All statistical analyses were performed using R version 4.3.3 and GraphPad Prism version 10.1.2. A p-value of < 0.05 was considered statistically significant for all analyses, and multiple testing corrections were applied where appropriate.

For comparisons between two groups (e.g., cytokine concentrations between MD patients and healthy controls), the non-parametric Wilcoxon rank-sum test (Mann-Whitney U test) was used. The Benjamini-Hochberg (BH) procedure was applied to adjust p-values for multiple comparisons. For comparisons across more than two groups (e.g., expression levels of hub genes across four patient groups), the non-parametric Kruskal-Wallis test was employed. When a significant difference was detected, Dunn’s *post-hoc* tests with Holm’s correction for multiple comparisons were applied. Paired t-tests were used before and after stimulation between groups. Analysis of variance was used for comparisons among multiple groups, followed by pairwise comparisons using the Bonferroni method. Correlations between variables (e.g., hub genes and immune cells) were assessed using Spearman’s rank correlation coefficient, with p-values adjusted using the BH method.

## 3 Results

### 3.1 Pathogen stimulation reveals a latent pro-inflammatory potential in MD PBMCs

**Figure 2:**
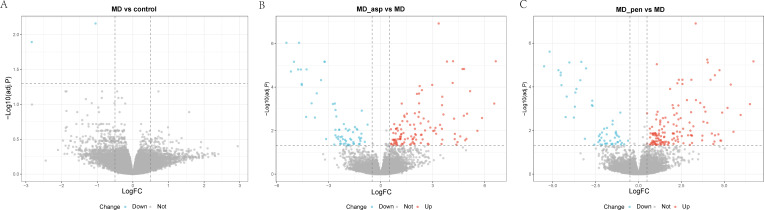
Volcano plot of DEGs in three comparison groups of GSE109558. **(A)** MD patients versus healthy controls; **(B)** Aspergillus-stimulated versus unstimulated MD; **(C)** Penicillium-stimulated versus unstimulated MD. Blue dots represent down-regulated genes, red dots up-regulated genes, and grey dots genes with no significant expression difference. DEGs, Differentially expressed genes; MD, Meniere’s disease; MD_asp, Aspergillus-stimulated Meniere’s disease; MD_pen, Penicillium-stimulated Meniere’s disease; adj P, adjusted P-value; logFC, log fold change.

To investigate the immune status of the remission phase of MD patients, we analyzed DEGs in three comparison groups. The first group compared MD patients with healthy controls. The second and third groups compared Aspergillus-stimulated and Penicillium-stimulated MD patients with unstimulated MD patients, respectively. The volcano plot (**Figure 2**) illustrates the results. Blue dots represent down-regulated genes, and red dots indicate up-regulated genes. When comparing MD patients with healthy controls, only two down-regulated genes were identified. No up-regulated genes were detected (**Figure 2A**). This suggests a relatively quiescent systemic immune state during the clinical remission phase. Aspergillus stimulation resulted in 117 up-regulated and 66 down-regulated genes (**Figure 2B**), while Penicillium stimulation led to 144 up-regulated genes and 58 down-regulated genes in the MD patients compared to their unstimulated group (**Figure 2C**). The markedly greater number of DEGs following fungal challenge, particularly the up-regulated genes after Penicillium stimulation, indicates a heightened responsiveness in PBMCs from MD patients to specific environmental triggers. It may reflects the molecular basis of the hyperinflammatory state observed during acute attacks.

### 3.2 Enrichment analysis highlights inflammatory pathways

**Figure 3:**
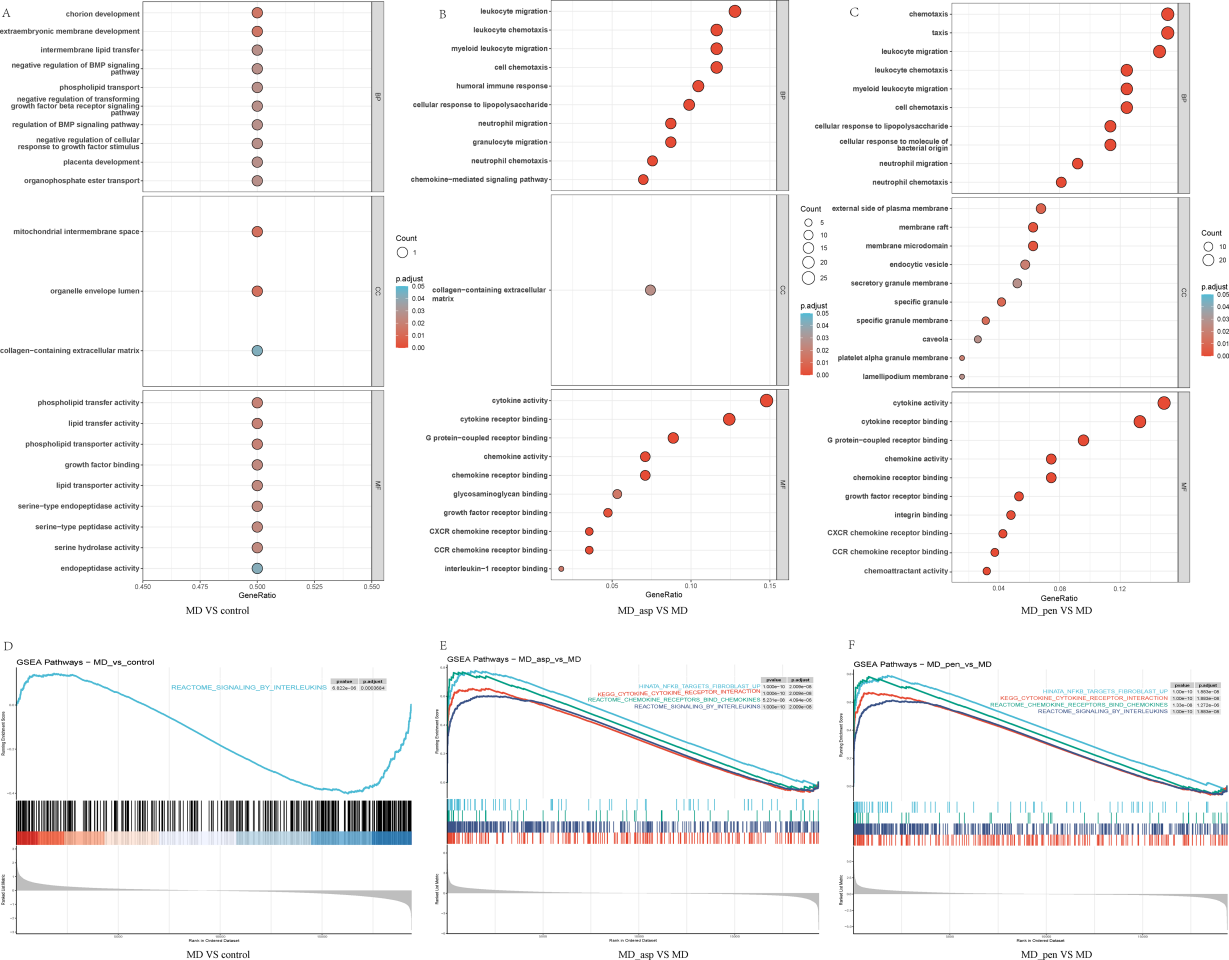
Enrichment Analysis of DEGs for the three key comparisons. MD vs Control: Results from GO **(A)** and GSEA **(D)** for the comparison of unstimulated MD patients versus healthy controls. MD patients in remission show slight enrichment in interleukin signaling pathways. MD_asp vs. MD: Results from GO **(B)** and GSEA **(E)** for the comparison of Aspergillus-stimulated versus unstimulated MD patient. Aspergillus exposure triggers a strong centered on leukocyte migration, chemotaxis, and NF-κB signaling. MD_pen vs. MD: Results from GO **(C)** and GSEA **(F)** for the comparison of Penicillium-stimulated versus unstimulated MD patient. Penicillium exposure similarly upregulates chemotaxis and immune response pathways, with notable enrichment in CXCL8-related and interleukin-10 signaling modules. GO, Gene ontology; GSEA, Gene set enrichment analysis; DEGs, Differentially expressed genes; MD, Meniere’s disease; MD_asp, Aspergillus-stimulated Meniere’s disease; MD_pen, Penicillium-stimulated Meniere’s disease; BP, Biological process; CC, Cellular Component; MF, Molecular Function.

Gene Ontology (GO) and Gene Set Enrichment Analysis (GSEA) were performed to investigate the biological functions in MD patients compared to healthy controls, and in MD patients before and after stimulation with Aspergillus or Penicillium. The top 10 enriched pathways for each group are displayed (**Figure 3**). In unstimulated MD patients, GSEA revealed significant enrichment of interferon response pathways (REACTOME_SIGNALING_BY_INTERLEUKINS).

Following Aspergillus stimulation, GO terms were dominated by leukocyte migration (e.g., leukocyte chemotaxis, neutrophil migration) and immune responses (e.g., humoral immune response, cellular response to lipopolysaccharide). GSEA further highlighted activation of pro-inflammatory and profibrotic mediator pathways (WP_OVERVIEW_OF_PROINFLAMMATORY_AND_PROFIBROTIC_MEDIATORS) and NF-κB signaling (HINATA_NFKB_TARGETS_FIBROBLAST_UP). These changes were accompanied by upregulation of genes associated with CXCL8-mediated chemotaxis, consistent with enhanced recruitment of neutrophils and other innate immune cells to the site of inflammation. In Penicillium-stimulated MD patients, GO analysis also emphasized leukocyte migration (e.g., neutrophil chemotaxis) and cellular responses to bacterial molecules. GSEA indicated enrichment in radiation stress response (GHANDHI_DIRECT_IRRADIATION_UP) and interleukin-10 signaling (REACTOME_INTERLEUKIN_10_SIGNALING). Notably, CXCL8-related pathways were enriched, underscoring the central role of this chemokine in orchestrating the innate immune response. Both fungal stimulation conditions showed overlapping enrichment in leukocyte migration, chemotaxis, and inflammatory response pathways, with CXCL8 serving as a key mediator of neutrophil recruitment. These findings suggest that pathogen exposure in MD patients triggers a robust innate immune response characterized by enrichment of transcriptional signatures associated with CXCL8-driven chemotaxis and inferred neutrophil infiltration.

### 3.3 PPI network identifies CXCL8 as a top hub inflammatory gene

**Figure 4:**
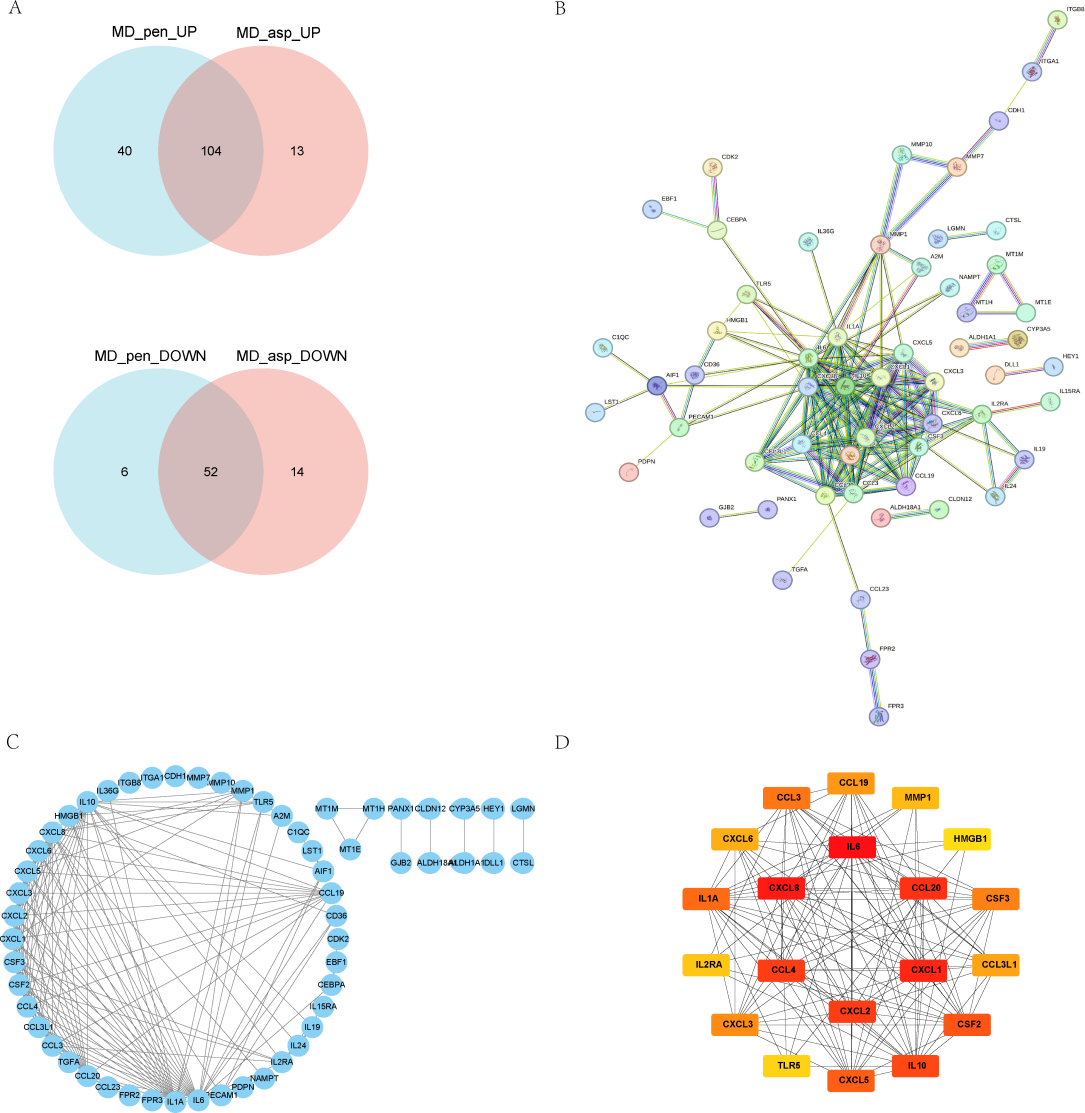
**(A)** Venn diagram showing DEGs in Aspergillus-stimulated PBMCs compared with MD, and Penicillium-stimulated PBMCs compared with MD. **(B)** PPI analysis and identification of hub genes for Aspergillus- and Penicillium-stimulated PBMCs in MD. The STRING database was used to predict interactions among the 105 up-regulated and 53 down-regulated common DEGs. **(C)** Network analysis using the Network Analyzer plugin in Cytoscape. **(D)** Identification of hub genes using the Cytohubba plugin and determination of the top 20 genes. Red = High up-regulation of gene expression, Orange = Moderate up-regulation, Yellow = Low up-regulation or mild expression change. MD, Meniere’s disease; MD_asp, Aspergillus-stimulated Meniere’s disease; MD_pen, Penicillium-stimulated Meniere’s disease.

**Figure 5:**
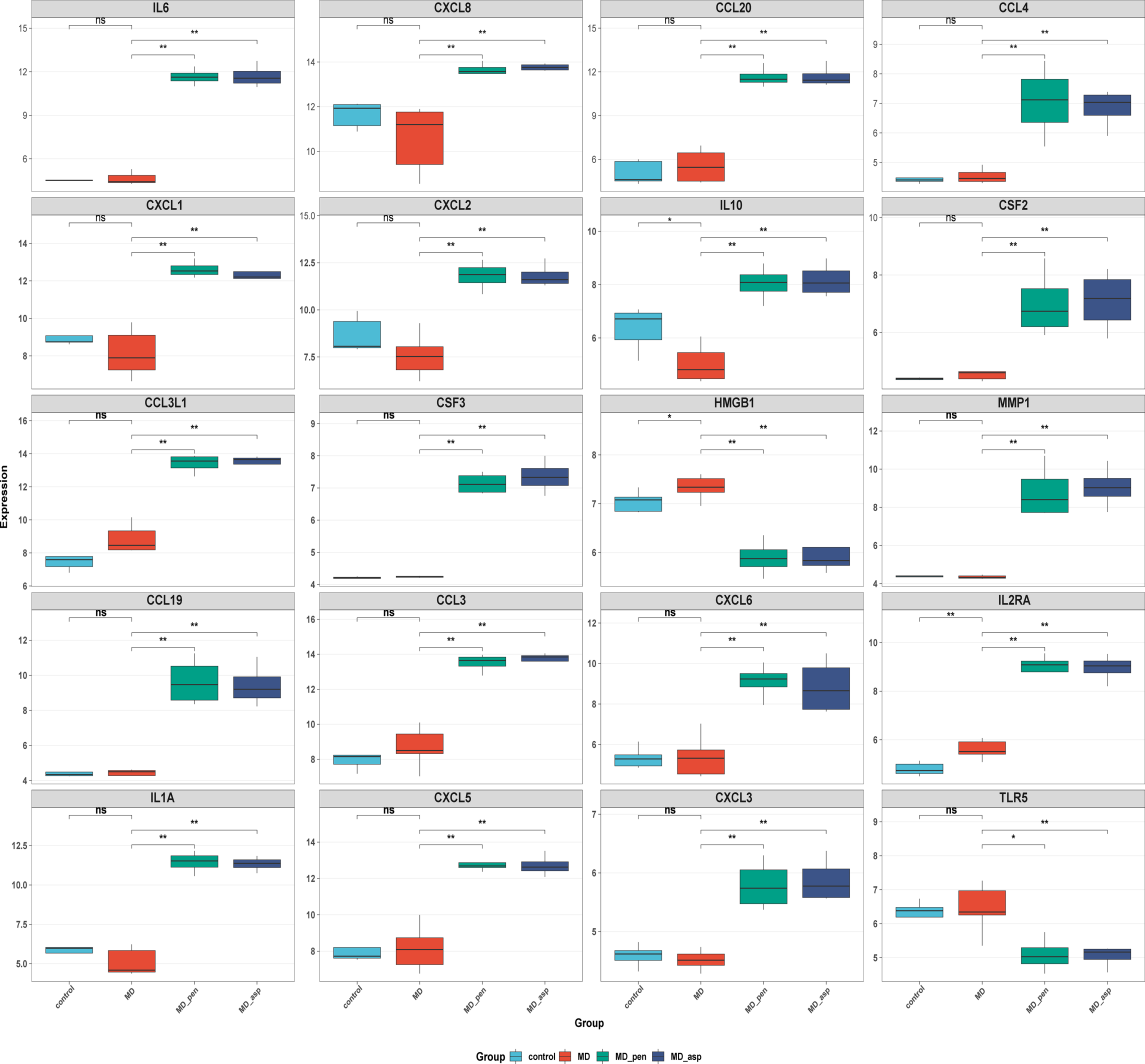
Expression of hub genes in four groups: healthy control, MD patients, and Aspergillus- and Penicillium-stimulated PBMCs in MD. Corrected significance markers indicate ns, not significant, *P<0.05, **P<0.01, ***P<0.001. In box plots, the horizontal line represents the median, the box indicates the interquartile range, and the whiskers indicate the minimum and maximum values excluding outliers. MD, Meniere’s disease; MD_asp, Aspergillus-stimulated Meniere’s disease; MD_pen, Penicillium-stimulated Meniere’s disease.

To pinpoint core regulators of the observed immune response, a PPI network was constructed using the STRING database. The network was based on co-expressed genes from the Aspergillus and Penicillium stimulation groups, including 104 up-regulated and 52 down-regulated DEGs (**Figure 4A**). The network consisted of 152 nodes and 170 edges (PPI enrichment P < 1.0×10^16^) (**Figure 4B**). Further analysis using the MCODE algorithm in Cytoscape identified two densely connected modules (cluster score > 3), from which 20 hub genes were selected (**Figures 4C, D**). These hub genes primarily function as pro-inflammatory factors (IL-6, CXCL8, CXCL1, CXCL2, CCL4, CCL20), immunoregulatory factors (IL-10, IL1A, IL2RA, TLR5), and extracellular matrix remodeling factors (MMP1, HMGB1). All hub genes exhibited significant differential expression in the MD group after stimulation with pathogen (**Figure 5**). Notably, CXCL8 emerged as a node with high connectivity, underscoring its potential central role.

### 3.4 Immune microenvironment analysis

**Figure 6:**
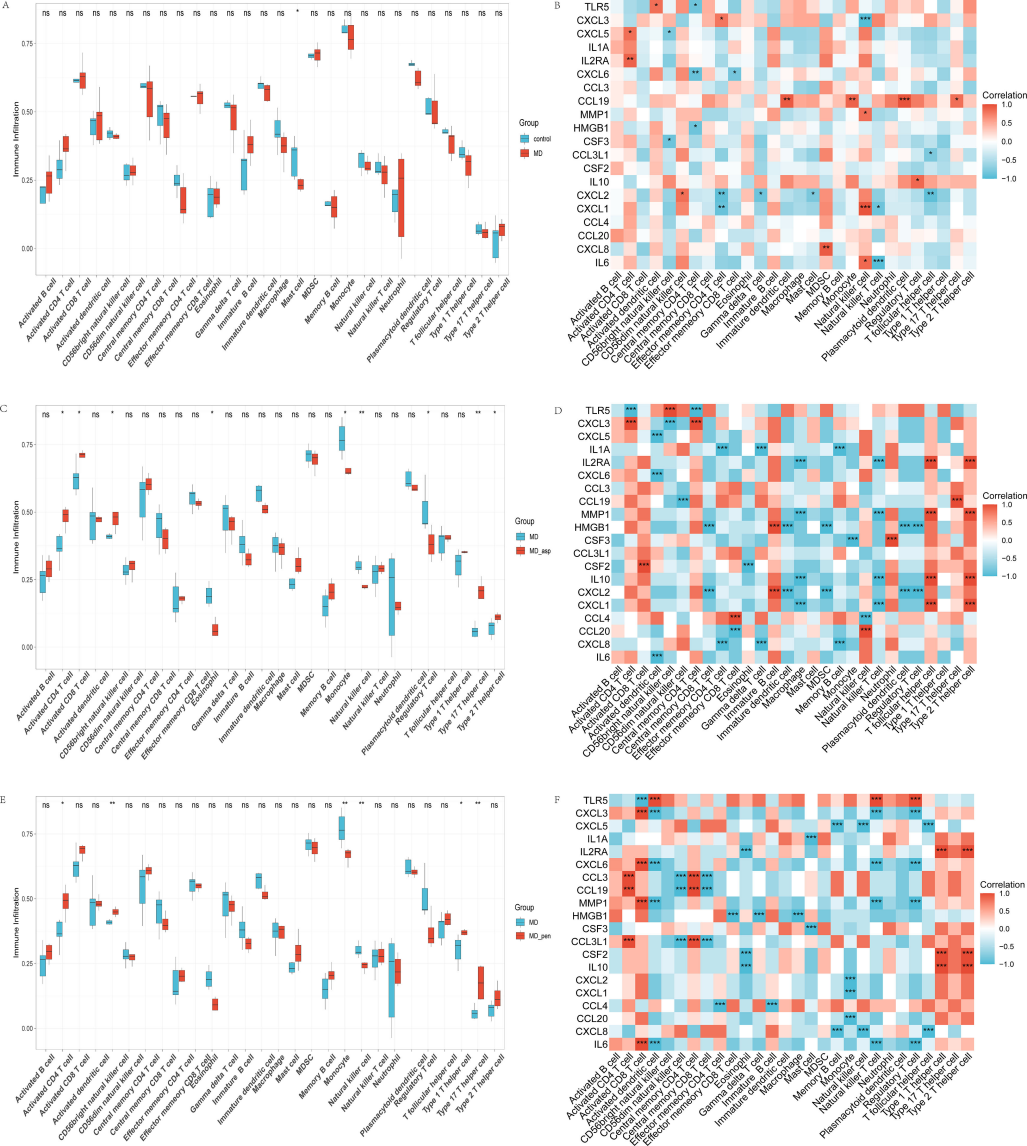
Grouped bar charts of immune infiltration results using the ssGSEA algorithm for three comparison groups: **(A)** MD vs healthy controls, **(C)** Aspergillus-stimulated vs MD, **(E)** Penicillium-stimulated vs MD. Correlation heatmaps between the 20 hub genes and immune cells in the three comparison group: **(B)** MD vs healthy controls, **(D)** Aspergillus-stimulated vs MD, **(F)** Penicillium-stimulated vs MD. MD, Meniere’s disease; MD_asp, Aspergillus-stimulated Meniere’s disease; MD_pen, Penicillium-stimulated Meniere’s disease. Corrected significance markers indicate ns, not significant, *P < 0.05, **P < 0.01, ***P < 0.001.

**Figure 7:**
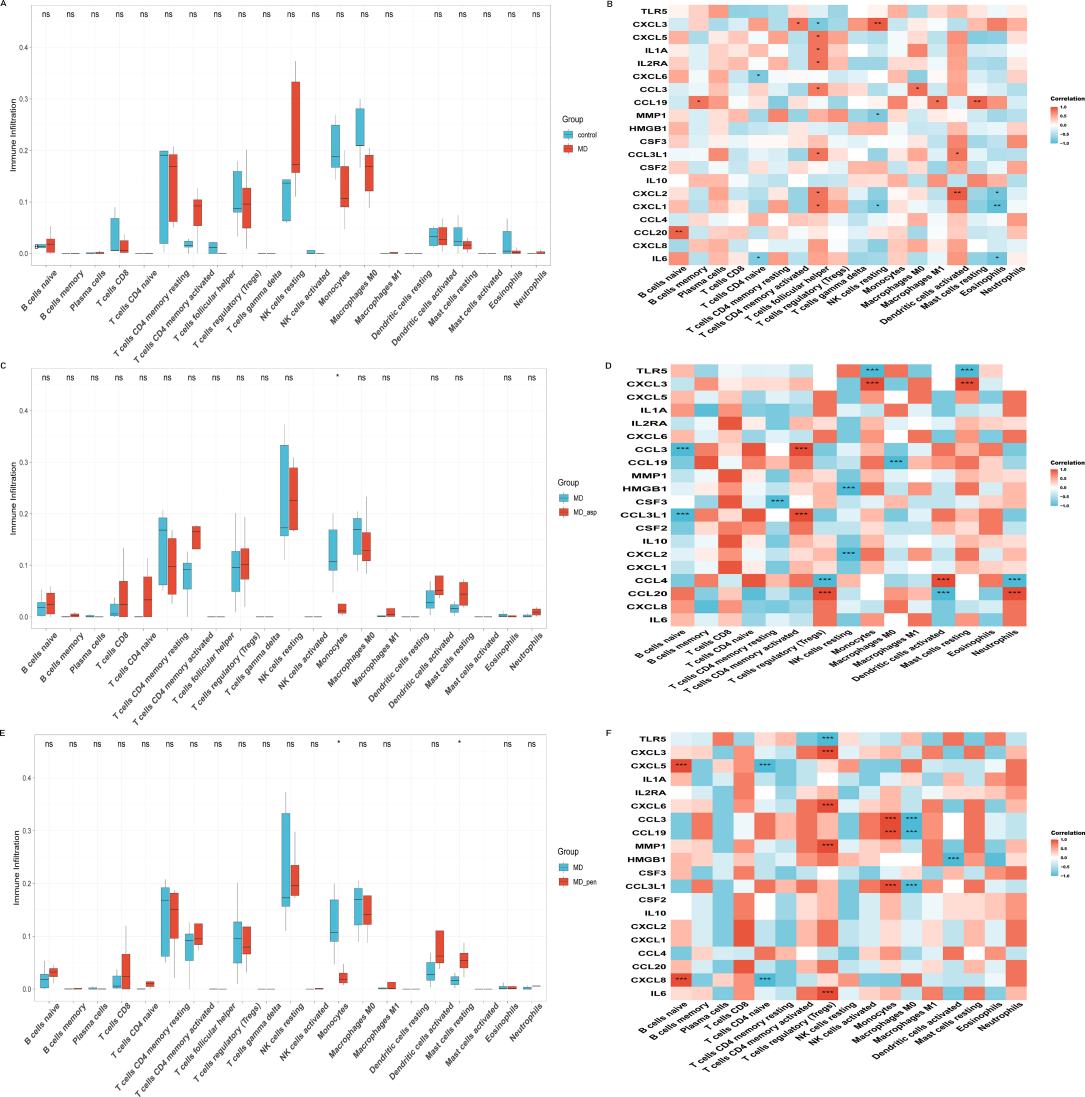
Grouped bar charts of immune infiltration results using the CIBERSORT algorithm for three comparison groups: **(A)** MD vs healthy controls, **(C)** Aspergillus-stimulated vs MD, **(E)** Penicillium-stimulated vs MD. Correlation heatmaps between the 20 hub genes and immune cells in the three comparison group: **(B)** MD vs healthy controls, **(D)** Aspergillus-stimulated vs MD, **(F)** Penicillium-stimulated vs MD. MD, Meniere’s disease; MD_asp, Aspergillus-stimulated Meniere’s disease; MD_pen, Penicillium-stimulated Meniere’s disease. Corrected significance markers indicate ns, not significant, *P < 0.05, **P < 0.01, ***P < 0.001.

We studied the immune microenvironment in various MD subgroups by applying two algorithms: ssGSEA and CIBERSORT. The ssGSEA analysis showed a significant reduction in mast cell infiltration in MD patients (P < 0.05) (**Figure 6A**). In contrast, CIBERSORT results indicated no significant differences in overall immune cell infiltration (**Figure 7A**). ssGSEA analysis indicated an inferred increase in the abundance of activated CD4^+^/CD8^+^ T cells and Th2/Th17 cells (P < 0.05) after Aspergillus stimulated. It also caused a decrease in eosinophils, monocytes, Tregs (P < 0.05), and NK cells (P < 0.01) (**Figure 6C**). In contrast, CIBERSORT analysis showed a decrease in monocytes only (P < 0.05) (**Figure 7C**). ssGSEA analysis suggested an increase in the proportion of activated CD4^+^ T cells (P < 0.05), CD56bright NK cells (P < 0.01), and Th1/Th17 cells (P < 0.05) after Penicillium stimulated. Meanwhile, monocytes and NK cells decreased (P < 0.01) (**Figure 6E**). CIBERSORT analysis revealed an increase in resting mast cells (P < 0.05) and a decrease in monocytes (P < 0.01) (**Figure 7E**).

In MD patients, ssGSEA analysis revealed several significant correlations. IL-6 was positively correlated with NK cells (P < 0.05). CXCL8 showed a positive correlation with myeloid-derived suppressor cells (MDSCs, P < 0.01). CXCL1 was negatively correlated with effector memory CD4^+^ T cells (P < 0.01) and positively correlated with NK cells (P < 0.001). IL-10 was positively correlated with Tregs (P < 0.05). Additionally, CCL19 was positively correlated with plasmacytoid dendritic cells (P < 0.001) and monocytes (P < 0.01) (**Figure 6B**). CIBERSORT analysis revealed several significant correlations. IL-6 was negatively correlated with naive CD4^+^ T cells (P < 0.001). CCL20 showed a positive correlation with naive B cells (P < 0.01). CXCL1 was negatively correlated with NK cells (P < 0.05). Additionally, CCL19 was positively correlated with resting mast cells (P < 0.01) (**Figure 7B**).

Following pathogen stimulation, most hub genes also showed significant correlations with immune cells. Analysis of ssGSEA in Aspergillus stimulation in MD patients showed that CCL4 was negatively correlated with Tregs (P < 0.001), CXCL1 and MMP1 were positively correlated with Th2/Tfh cells (P < 0.001), and TLR5 was positively correlated with CD56^bright^ NK cells (P < 0.001) (**Figure 6D**). CIBERSORT results showed that CCL20 was positively correlated with Tregs and neutrophils (P < 0.001). CXCL3 was positively correlated with monocytes (P < 0.001), whereas TLR5 was negatively correlated with monocytes (P < 0.001) (**Figure 7D**). Similarly, ssGSEA analysis of Penicillium stimulation in MD patients revealed that IL-6 was positively correlated with activated CD8^+^ T cells (P < 0.001), CXCL8 was negatively correlated with memory B cells (P < 0.001), IL-10 and CSF2 were positively correlated with Th1/Th2 polarization (P < 0.001), and CCL3L1, CCL3, and CCL19 were positively correlated with activated CD4^+^ T cells (P < 0.001) (**Figure 6F**). CIBERSORT results further revealed positive correlations of IL-6 and CXCL6 with Tregs (P < 0.001), CXCL8 with naive B cells (P < 0.001), and CCL3L1 and CCL3 with monocytes (P < 0.001) (**Figure 7F**).

### 3.5 Serum cytokine profiling confirms systemic inflammatory surge during acute attacks

**Figure 8:**
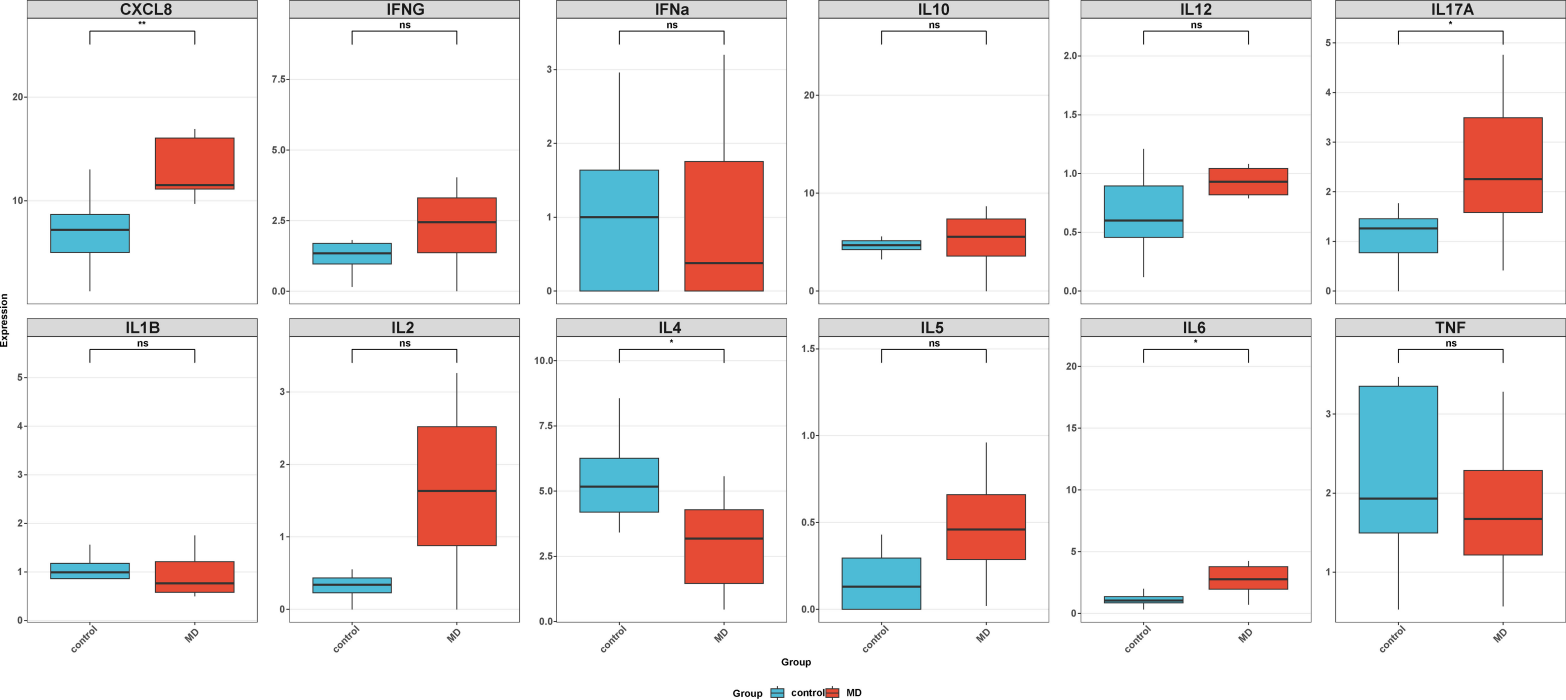
The expression of serum cytokine at the onset of MD. ns, not significant; *P < 0.05, **P < 0.01, ***P < 0.001. MD, Meniere’s disease.

To translate *in vitro* findings into the *in vivo* context, we measured serum cytokine levels in an independent cohort of MD patients during acute attacks. Consistent with the hyperinflammatory hypothesis, we observed elevated serum cytokine levels during these episodes in MD patients. In Meniere patients, the expression levels of CXCL8, IL17A, and IL6 were significantly increased during acute attacks compared to healthy individuals. The expression of IL4 was significantly decreased (**Figure 8**). This pattern validates the systemic pro-inflammatory shift detected in our bioinformatic analysis during symptomatic episodes.

### 3.6 Single-cell analysis positions macrophages as the primary source of CXCL8

**Figure 9:**
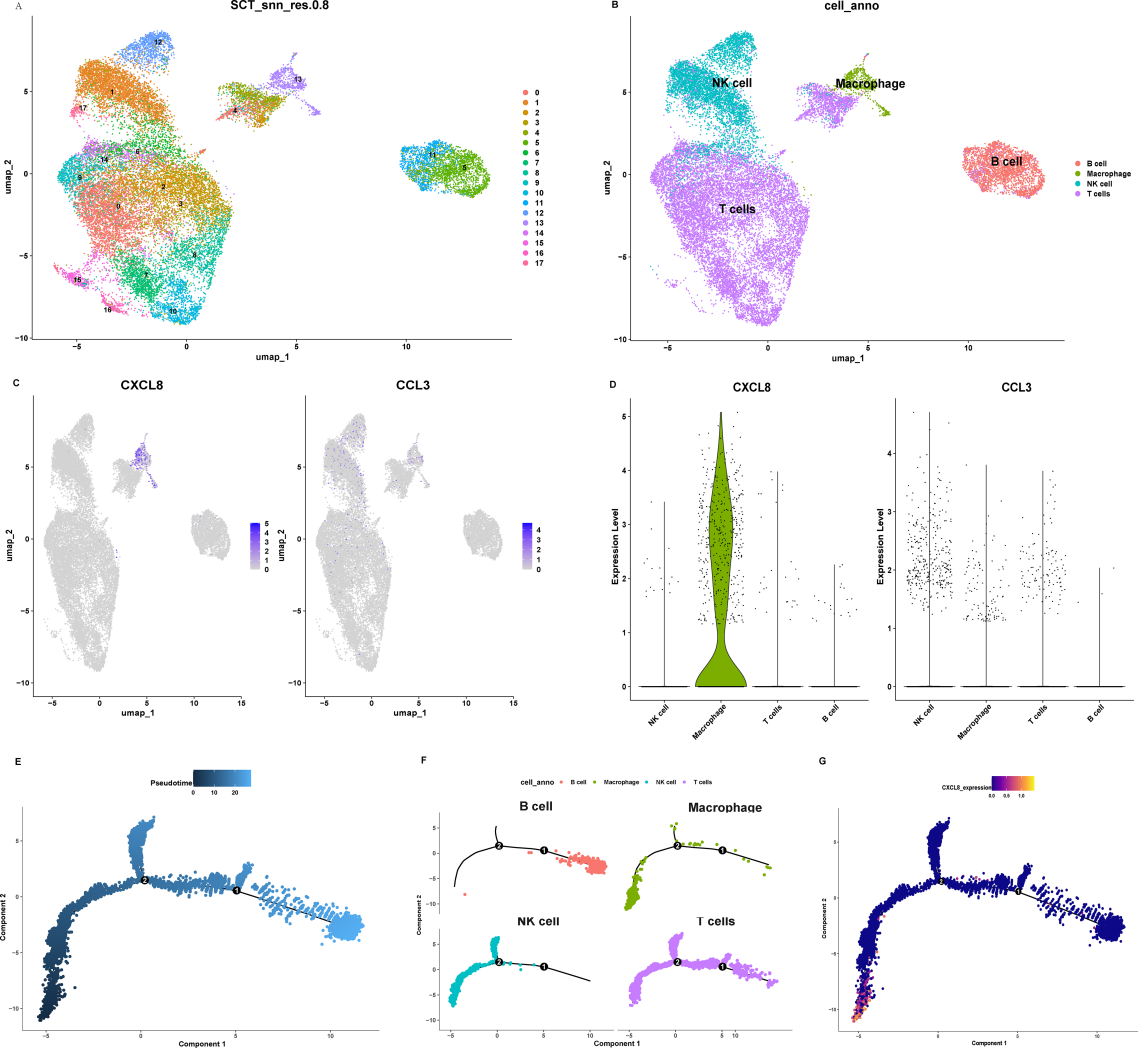
Single-cell transcriptome landscape of MD. **(A)** UMAP of single-cell clusters with a resolution of SCT_snn_res.0.8, **(B)** UMAP visualization of single-cell clusters labeled by cell types. **(C)** The distribution of hub genes in cell clusters. **(D)** The expression levels of hub genes in different cell types. **(E)** Pseudotime trajectories of single cells in MD. **(F)** The situation of different cell types in pseudo-time trajectories. **(G)** The pseudo-timing trajectory of CXCL8. Color intensity indicates the relative expression level. MD, Meniere’s disease; UMAP, Uniform manifold approximation and projection.

We analyzed the single-cell RNA sequencing data of MD PBMCs and explored the expression of core genes IL6, IL10, IL17A, CCL3, CCL19, CXCL1, CXCL8, CCL20, CXCL12 and IL4 among them. Unsupervised clustering identified 17 distinct clusters, which were annotated into major immune lineages: B cells, T cells, NK cells, and macrophages (**Figures 9A, B**). Among the core genes, only CXCL8 and CCL3 showed differential expression. It revealed that CXCL8 was predominantly expressed by a subset of macrophages (**Figures 9C, D**). Pseudotime reconstruction uncovered a branched differentiation trajectory with macrophages identified as the cellular origin, which could be further divided into three distinct phases (**Figures 9E, F**). In the early phase, macrophage subsets expressing high levels of CXCL8 dominated, accompanied by active proliferation of NK cells. This formed the primary inflammatory core driving the initial immune response. This was followed by T-cell and B-cell activation phases. This analysis nominates macrophages as the key initiators of the inflammatory cascade via CXCL8 production, providing a cellular mechanism for the observed hyperinflammatory response. Notably, CXCL8 emerged as an early checkpoint regulator in this trajectory (**Figure 9G**). Its expression was upregulated within the first of pseudotime and the peak expression of CXCL8 showed a significant correlation with the commitment of macrophages to inflammatory states, highlighting its pivotal role in initiating and shaping the inflammatory trajectory.

**Figure 10:**
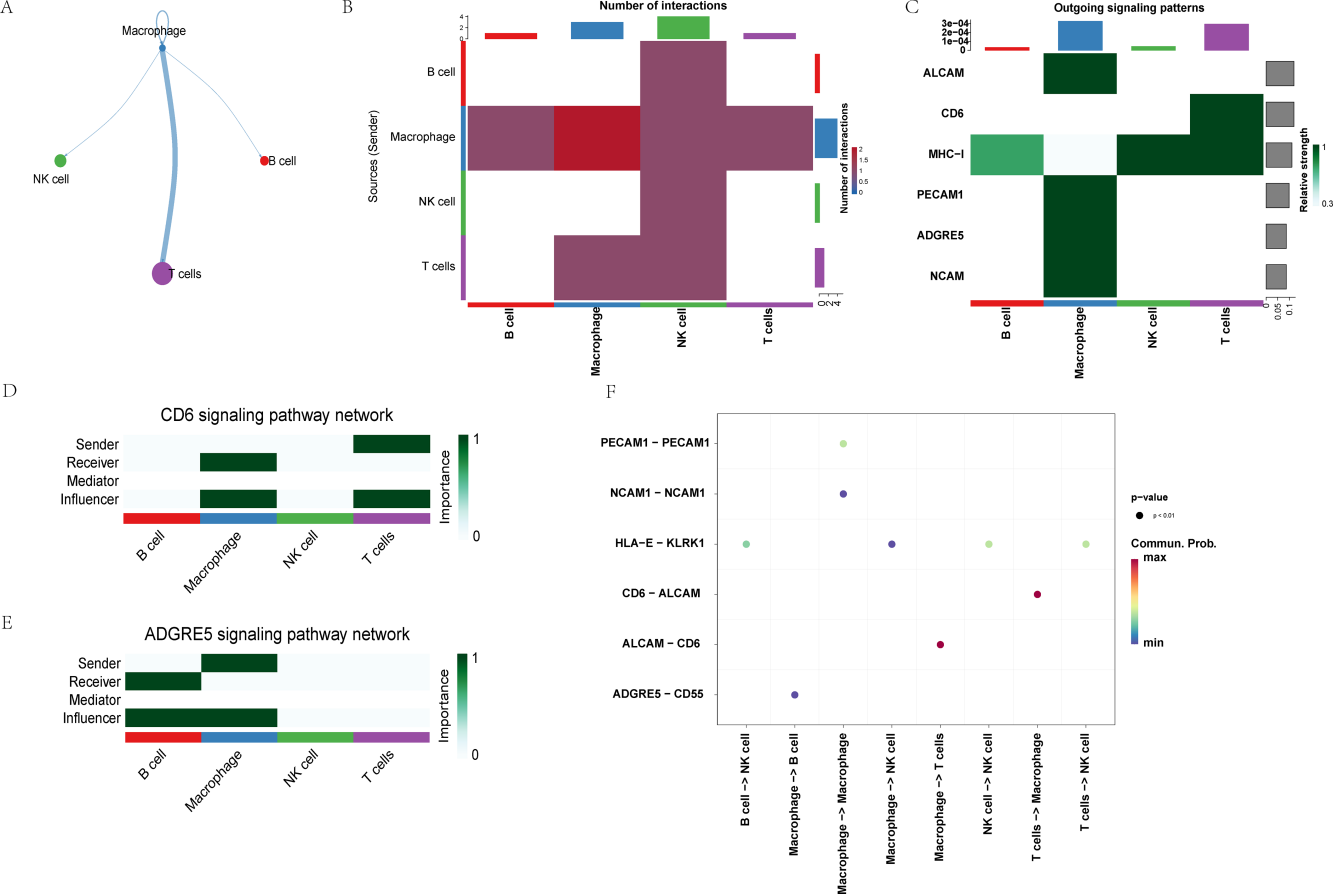
Cellchat analysis of single-cell sequencing in MD. **(A)** The cellchat network of macrophages. **(B)** Heat map of intercellular communication. **(C)** The outgoing signaling heatmap of the different cell types. The central heatmap signaling pathways of cellchat, CD6 signaling pathway **(D)** and ADGRE5 signaling pathway **(E)**. **(F)** Interaction of the different cell types in MD. MD, Meniere’s disease.

CellChat analysis identified macrophages as dominant signaling hubs with dual roles as both senders and influencers of intercellular communication. Macrophages primarily signal with T cells (**Figures 10A, B**), and the ALCAM→CD6 interaction dominates the communication between the two cell types (**Figure 10F**). Specifically, in the CD6 pathway, macrophages functioned as both influencers and receivers (**Figure 10D**). In the ADGRE5 (CD97) pathway, macrophages acted as both senders and influencers (**Figure 10E**). Macrophages also served as primary senders in the ALCAM pathway and the PECAM1 pathway (**Figure 10C**). Cell communication analysis suggests macrophages may influence adaptive immunity through ALCAM–CD6 signaling pathways.

### 3.7 Functional validation macrophages as a key source of CXCL8 upon innate immune stimulation

**Figure 11:**
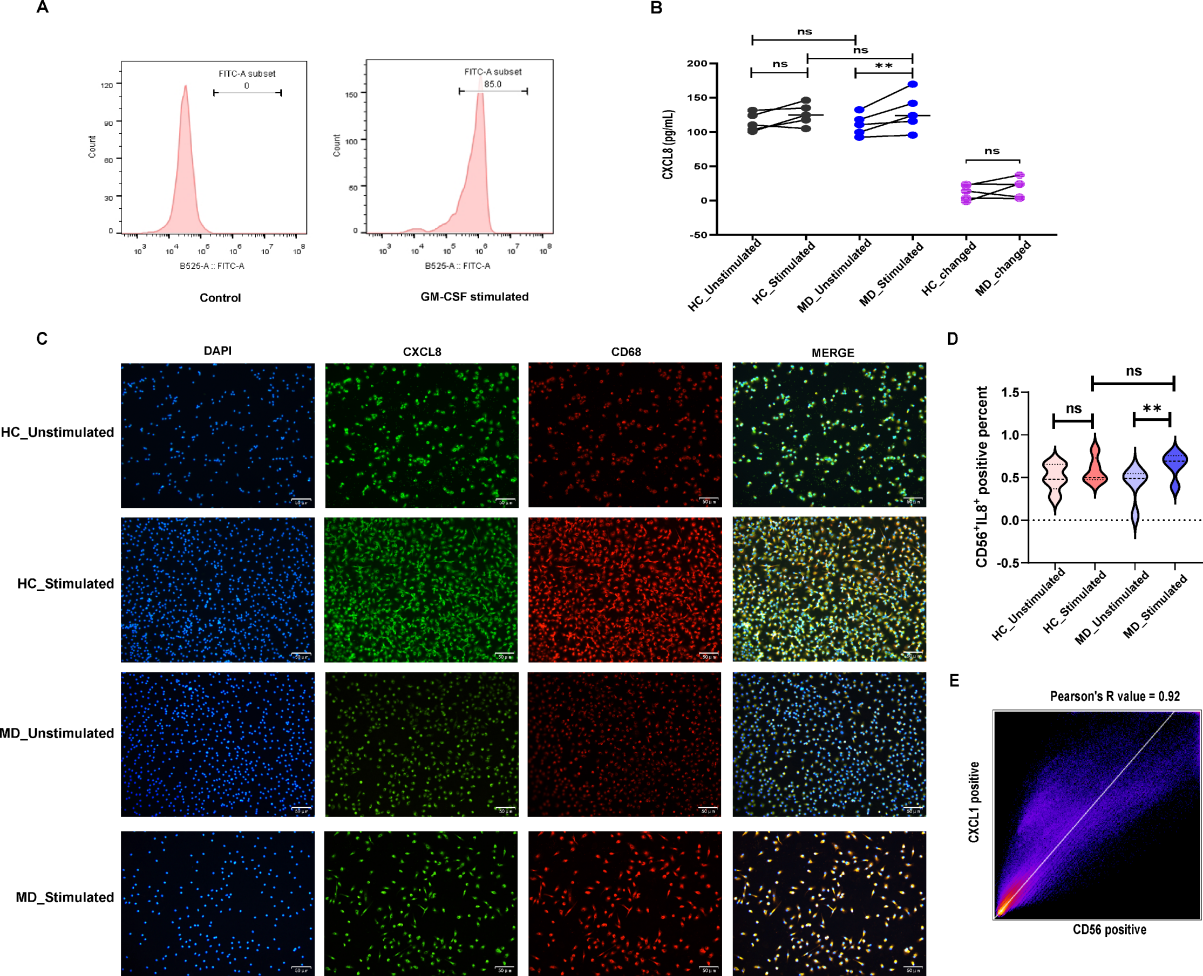
In vitro validation of CXCL8 secretion and expression in monocyte-derived macrophages. **(A)** Flow cytometry verification of induction effectiveness. After 24 hours of combined stimulation, **(B)** ELISA was used to determine the concentration of CXCL8 in the cell culture supernatant; **(C)** Result of immunofluorescence; **(D)**Representative immunofluorescence images of co-expression of CD68 (red) and CXCL8 (green) in macrophages. The cell nuclei show blue fluorescence at a wavelength of 385nm. HC_unstimulated: healthy control treated without stimulated; HC_unstimulated: healthy control treated with compound stimulation(100 ng/mL lipopolysaccharide and 10 μg/mL β-glucan); MD_unstimulated: Meniere’s disease treated without stimulated; HC_unstimulated: Meniere’s disease treated with compound stimulation(100 ng/mL lipopolysaccharide and 10 μg/mL β-glucan). ns, not significant; **P < 0.01.

To functionally validate the role of macrophages and CXCL8 identified in our bioinformatic and single-cell analyses, we isolated PBMCs from acute attack of MD patients and healthy controls, differentiated them into macrophages *in vitro* (**Figure 11A**). After *in vitro* stimulation, CXCL8 levels measured by ELISA significantly increased compared to the unstimulated baseline in MD attacked patients (p < 0.01). In contrast, macrophages from healthy controls (HC) showed no significant response to the same stimulus (**Figure 11B**). While the absolute CXCL8 levels post-stimulation and the magnitude of change did not differ significantly between the MD and HC groups, the fundamental qualitative difference lies in the responsiveness itself. The fact that a significant CXCL8 release was triggered in MD macrophages, but not in HC macrophages, by the same stimulus intensity reveals a lower activation threshold and a pathological hyperreactivity in MD.

To confirm that this response originated from macrophages themselves, we performed immunofluorescence (IF) staining for the macrophage marker CD68 and CXCL8 (**Figure 11C**). Semi-quantitative analysis of the IF images directly corroborated the ELISA findings. The percentage of CD68^+^ double-positive macrophages was significantly higher in the stimulated MD group compared to its unstimulated control (p < 0.01) (**Figure 11D**). Pearson’s correlation analysis confirmed strong colocalization of CXCL8 with CD68 signals (Person value > 0.7), verifying the cellular source (**Figure 11E**). Again, no significant increase in double-positive cells was observed in the HC group following stimulation.

## 4 Discussion

**Figure 12:**
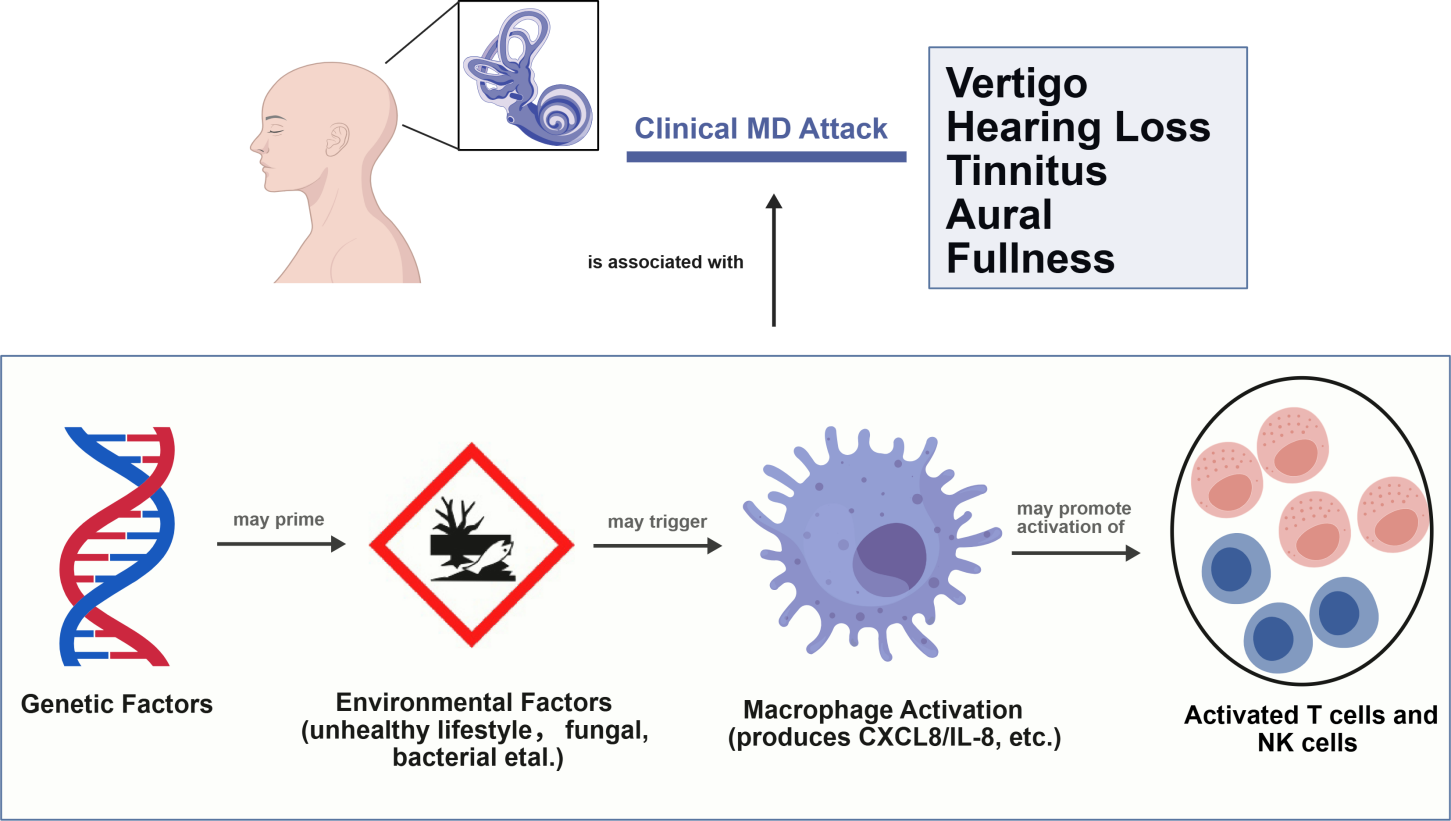
A proposed immunopathological mechanism in MD. This model integrates transcriptomic findings from pathogen-stimulated PBMCs, single-cell RNA-seq analysis, and in vitro experiments. It proposes that in individuals with genetic factors, environmental factors (e.g., unhealthy lifestyle, fungal, bacterial et al.) may activate macrophages, leading to the production of pro-inflammatory mediators such as CXCL8/IL-8. This response may subsequently promote the activation of T cells and natural killer (NK) cells. That maybe associated with the attack of MD. Whether this specific immune sequence directly triggers an acute attack of MD still requires further research to determine. (Draw using BioGDP).

The treatment goal of MD is to prevent or reduce the severity and frequency of vertigo. It also aims to alleviate or prevent hearing loss, tinnitus, and ear fullness. Ultimately, the goal is to improve the quality of life for patients. Treatment focuses not only on managing acute attacks. It also involves making long-term changes in lifestyle and diet to reduce future problems (1, 2). This shows that MD is more than just a disease with alternating attack and recovery phases. In our study, results from DEGs showed that MD patients have more stable gene activity during asymptomatic periods. This might indicate that their immune system appears normal. However, after exposure to pathogens, the number of active genes significantly increased. This suggests that the immune system in MD patients becomes overly reactive. Our findings reveal a dysregulated immune response in MD characterized not by constitutive hyperactivation, but by a pathological hyperreactivity to external triggers. The absence of baseline differences between patients and controls during the quiescent phase aligns with the clinical ‘hypoimmune’ phenotype. However, the exaggerated CXCL8 release upon challenge uncovers a latent ‘hyperinflammatory’ potential. This pattern contrasts with a previous report by Sun et al., which identified 366 DEGs in MD patients. The considerable discrepancy likely stems from differences in patient selection criteria. Sun et al. focused exclusively on patients with unilateral MD (type 1) (30), while the cohort used in our primary bioinformatic analysis (GSE109558) consisted of patients in the clinically quiescent phase, without restriction to laterality or specific disease subtype. This distinction underscores the critical influence of clinical heterogeneity on transcriptional profiles and highlights that the hyperreactive immune phenotype in MD may be most evident upon provocation rather than at baseline. This study provides a multi-faceted exploration of the immune mechanisms in MD, combining bioinformatic analyses of pathogen-stimulated PBMCs, analyzed at single-cell resolution, with experimental validation. Our findings consistently support a paradigm of a “hypoimmune-hyperinflammatory switch”, which was summarized in **Figure 12** (Draw using BioGDP) (31). In this model, a seemingly quiescent immune state during clinical remission of MD masks a latent hyper-reactivity, which can be unleashed by environmental triggers; and ultimately leading to acute inflammatory attacks.

We found a stark contrast in transcriptional profiles between unstimulated and pathogen-stimulated PBMCs from MD patients. The little difference in gene expression in unstimulated cells aligns with the clinical observation of a “low-cytokine phenotype” during remission (8) and suggests systemic immune quiescence. However, exposure to Aspergillus or Penicillium antigens revealed a profound latent inflammatory capacity. This was characterized by the significant upregulation of genes involved in neutrophil chemotaxis and NF-κB signaling. This reflects clinical exacerbations linked to allergies or infections (1, 10) and provides a molecular basis for the hyperinflammatory state we propose. This matches earlier studies showing that immune system problems are key to the development of MD (32, 33). The large number of genes related to tissue development may indicate aberrant remodeling in the inner ear, potentially linked to endolymphatic hydrops (EH), a major feature of MD (1).

We identified 20 hub genes involved in the immune problems of MD. These genes fall into three categories: pro-inflammatory cytokines such as IL-6, CXCL8/IL-8, and CXCL1; immune regulators like IL-10 and TLR5; and factors affecting tissue remodeling, including MMP1 and HMGB1. Consistent with prior evidence (7, 33), elevated IL-6 and CXCL8 promote neutrophil recruitment and vascular hyperpermeability. We also found high expression of IL8/CXCL8 in the peripheral blood of patients during the MD attack period. The central finding of our functional validation is not the quantitative difference in cytokine output, but the qualitative difference in responsiveness. The fact that a significant CXCL8 release was triggered in MD macrophages, but not in HC macrophages, by the same stimulus intensity suggests a lower activation threshold in the pathological state, indicating a fundamental dysregulation in immune signaling pathways in MD. The role of neutrophils in MD is also shown by CXCL1 and CXCL2, which are chemokines carried by neutrophils. These findings support the idea that NETs could lead to the immune system attacking inner ear tissues (33). The changes in IL-10 (an anti-inflammatory cytokine) and TLR5 (a receptor for pathogens) suggest dysregulation in pathways controlling inflammation and responding to pathogens, which may correlate with the clinical observation of heightened infection susceptibility in MD patients (3). MMP1 and HMGB1, which are linked to tissue damage and inflammation, may play a role in the development of EH by damaging the inner ear barrier (34). These hub genes show a pattern where infections can make inflammation worse, cause tissue damage, and trigger autoimmune responses. This fundamental alteration in immune response threshold may explain why MD attacks are often precipitated by seemingly benign environmental factors in susceptible individuals.

Our immune infiltration analysis revealed dynamic shifts in the MD immune landscape. Patients in the quiescent phase of MD showed reduced mast cell infiltration, aligning with studies where mast cell reactions cause MD-like symptoms in guinea pigs (35). After stimulation, both Aspergillus and Penicillium increased T-cell inflammation and decreased monocyte numbers. The inferred reduction in circulating monocytes suggests their potential recruitment to inflammatory sites, such as the inner ear, as seen in tissue studies of MD patients’ ES (7).

Our scRNA-seq analysis of PBMCs from patients with MD identified 17 distinct immune subpopulations, predominantly comprising B cells, macrophages, natural killer (NK) cells, and T cells. Among these, macrophages emerged as key orchestrators of inflammatory cascades. This finding is consistent with previous reports of macrophage infiltration in endolymphatic sac tissues (7). The pseudo-time trajectory identified CXCL8 as an early regulatory checkpoint at Stage I, driving neutrophil and NK cell activation and initiating a three-phase inflammatory cascade. Macrophage-dominated initiation, where CXCL8 upregulation triggers innate immune recruitment. Our immunofluorescence validation further confirmed that CXCL8 expression originates specifically from macrophages. Single-cell analysis provides direct evidence of the cellular source of CXCL8. While, our ssGSEA analysis complements this by revealing functional associations between CXCL8 and immune cell abundance in the microenvironment.CXCL8 can recruit and regulate the function of MDSCs (36), and may also promote B cell activation by affecting the migration and activation of dendritic cells (37). In stage II, T-cell effector activation occurs, mediated by ALCAM–CD6 co-stimulation and aligning with findings of Th1/Th17 polarization in MD autoinflammation (4). In stage III, terminal B-cell differentiation occurs, reflecting the existence of humoral immunity in MD. CellChat analysis revealed that macrophages control inflammation through main synergistic pathways including ALCAM-CD6 axis and ADGRE5 (CD97) pathway. It has been confirmed that macrophage-derived ALCAM binds to the T cell CD6 to form immune synapses between macrophages and T cells. This interaction amplifies the production of IL-6 and CCL20, and promotes Th17 polarization (38). Perhaps this mechanism also exists in Meniere’s disease, contributing to its pathogenesis. CD97 enhances CXCL8-mediated chemotaxis and sustains inflammation through self-reinforcing signals. This mechanism explains the chronicity of MD attacks. This dual mechanism supports the “double-hit” model of MD, where genetic susceptibility [e.g., NLRP3 variants (14)] interacts with environmental triggers such as fungal exposure. Notably, CXCL12 was included in the hub gene analysis. However, it did not show significant differential expression in our single-cell analysis and is therefore not discussed as a central player. The limitations of this study arise from several factors. First, the immune system is complex. MD has many causes, making the study challenging. The multi-pathogen stimulation model simulates environmental triggers. However, its applicability may be limited due to the specific pathogens selected and their distinct interactions with the immune system. Second, the *in vitro* stimulation model may not fully capture the complexity of *in vivo* environmental exposures. The observed difference in responsiveness, in the absence of a significant difference in the magnitude of change, may point to altered response kinetics in MD. It is possible that MD macrophages mount a more rapid response to challenge. The lack of a significant difference in the value could be attributed to the high heterogeneity among MD patients or differences in response kinetics, which future studies with larger cohorts and longitudinal designs should explore. The lack of detailed clinical staging metadata in the public single-cell dataset limited our ability to correlate transcriptional states with specific disease phases. The relatively small sample size of our independent validation cohort may also limit the statistical power for detecting subtle effects. Future studies incorporating spatial transcriptomics of human endolymphatic sac tissues, utilizing animal models of MD, or developing more complex multicellular culture systems could help better delineate inflammatory dynamics within the inner ear and further validate the therapeutic potential of targeting the macrophage-CXCL8 axis. Nonetheless, this demonstrable hyper-responsiveness aligns with the clinical picture of attacks being triggered by environmental factors and supports the core of our ‘switch’ hypothesis.

## 5 Conclusions

In conclusion, our study combining multi-omics and experiments provides strong evidence for a macrophage-centered immunopathological axis in MD. We propose a “hypoimmune-hyperinflammatory switch” model, where remission features relative immune quiescence interrupted by exaggerated inflammatory responses to environmental triggers. A key part of this model is the dysregulated function of macrophages, identified in our study as the primary source of the chemokine CXCL8. Immune responses mediated by pathways such as ALCAM-CD6 reveal a critical connection between innate immune activation and adaptive T-cell polarization. Building on this, our functional validation confirms that macrophages from MD patients possess an intrinsic hyperreactivity characterized by heightened cytokine production. These findings significantly advance our understanding of MD pathogenesis, moving beyond the observation of inflammation to delineate a specific cellular and molecular cascade. Targeting the macrophage-CXCL8 axis may provide a promising strategy for novel immunomodulatory treatments to prevent or lessen acute attacks in this disorder.

## Data Availability

The original contributions presented in the study are included in the article/**Supplementary Material**. Further inquiries can be directed to the corresponding author.
