# Supplementary material for: Macrophage-derived CXCL8 as a mediator of inflammatory attacks in Meniere’s disease
Source: Front Immunol. 2026 Mar 6;17:1683879. doi: 10.3389/fimmu.2026.1683879 (PMC13002460; doi:10.3389/fimmu.2026.1683879)
Supplement: Supplementary file 1 [file Table1.docx]

Supplementary Material

# Supplementary Figures and Tables

- 1. **Supplementary Tables**

| TableS1 Information of independent cohort | | | | | |
| --- | --- | --- | --- | --- | --- |
| Patient | Age (Year) | Sex | Laterality | Ear | Usable hearing (AAO-HNS) |
| 1 | 70 | FeMale | Unilateral | Right | C |
| 2 | 67 | Male | Unilateral | Left | C |
| 3 | 32 | FeMale | Unilateral | Left | B |
| 4 | 45 | FeMale | Unilateral | Right | B |
| 5 | 36 | FeMale | Unilateral | Left | A |
| 6 | 53 | Male | Unilateral | Left | B |
| 7 | 50 | Male | Unilateral | Left | B |
| 8 | 38 | FeMale | Bilateral | Bilateral | C |
| 9 | 51 | Male | Unilateral | Left | D |
| 10 | 57 | Male | Unilateral | Right | C |
| 11 | 58 | FeMale | Unilateral | Left | C |
| 12 | 73 | FeMale | Unilateral | Right | C |
| 13 | 23 | FeMale | Bilateral | Bilateral | C |
